# Effects of Melatonin on Early Pregnancy in Mouse: Involving the Regulation of *StAR*, *Cyp11a1*, and *Ihh* Expression

**DOI:** 10.3390/ijms18081637

**Published:** 2017-07-27

**Authors:** Shengyu Guan, Lu Xie, Teng Ma, Dongying Lv, Wang Jing, Xiuzhi Tian, Yukun Song, Zhiping Liu, Xianghong Xiao, Guoshi Liu

**Affiliations:** 1 College of Wildlife Resources, Northeast Forestry University, No. 26, Hexing Road, Xiangfang District, Harbin 150036, China; gsy729@hotmail.com (S.G.); liuzhp2000@163.com (Z.L.); hzy0106@gmail.com (X.X.); 2 National Engineering Laboratory for Animal Breeding, Key Laboratory of Animal Genetics and Breeding of the Ministry of Agriculture, Beijing Key Laboratory for Animal Genetic Improvement, College of Animal Science and Technology, China Agricultural University, Beijing 100193, China; luxiecau@163.com (L.X.); mateng7777@126.com (T.M.); suxingdemogu@icloud.com (D.L.); caylajing@cau.edu.cn (W.J.); tian7550@163.com (X.T.); songyukun@cau.edu.cn (Y.S.)

**Keywords:** melatonin, mice, early pregnancy, corpus luteum, E_2_, P

## Abstract

To test whether melatonin plays an important role in the process of early pregnancy, melatonin was given in drinking water to pregnant mice at different gestation stages. These included mice who were given melatonin 14 days prior to their successful mating (confirmed by vaginal plug) (Group A), after successful mating (Group B), and 14 days prior to and until after successful mating (Group C). Melatonin administration significantly enhanced serum as well as ovarian melatonin levels in the mice. It was observed that melatonin did not affect the natural estrous of mice. On day 0.5 of gestation (D0.5), melatonin not only elevated progesterone (P) secretion, but also upregulated expressions of *StAR* and *Cyp11a1*, the two marker genes of corpus luteum in ovaries (*p* < 0.05). Group A had a significantly lower estradiol (E_2_) secretion and a higher number of implantation sites as well as litter size than controls (*p* < 0.05) and also had an increased *Ihh* expression in endometrium of D7.5 gestation. Melatonin treatment after successful mating improved the progesterone (P) secretion at D7.5 of gestation (*p* < 0.05) and significantly induced leukaemia inhibitory factor (LIF) expression (*p* < 0.05). Our study indicates that melatonin treatment up-regulated the genes involved in pregnenolone synthesis in ovary and *Ihh* expression in uterine endometrium. The mechanisms of melatonin to improve embryo implantation related to their actions on promoting the development of corpus luteum before gestation and helping to specify uterine receptivity in early pregnant mice.

## 1. Introduction

In mammals, reproduction is a complex process with several critical steps: sexual maturity, estrus, early embryo development, embryo implantation, and post implantation fetal development [1]. Many factors influence this process including ovarian hormones, cytokines, growth factor, homeotic proteins, and morphogens [2]. Among them, melatonin (MT) is believed to play a critical role in animal reproductive physiology. Melatonin, the major pineal secretory product, is an amphiphilic molecule which readily enters all subcellular compartments including membrane, cytosol, nucleus, and mitochondrion. Melatonin is widely distributed in almost any tissue, organ, and cell [3,4]. It participates in multiple physiological processes [4,5,6]. It regulates the secretion of gonadotropin in the hypothalamus through the hypothalamus–pituitary–gonadal (HPG) axis, thus, influencing biorhythms, sexual maturation, seasonal estrus, reproductive behavior, and gamete protection [6,7,8,9,10,11]. Pévet and Haldar-Misra [12] reported that injection or oral melatonin had the same effect in the suppression of gonadal function of the golden hamster, and oral melatonin effectively inhibited the endogenous activity of the ovary [13]. After pinealectomy, rats show an increase in estradiol (E_2_) and a decrease in the progesterone (P) levels during the estrous phase [14].

As a powerful antioxidant, melatonin has the ability to scavenge reactive oxygen species (ROS) and reactive nitrogen species (RNS) via receptor-independent actions [15,16,17]. In addition, melatonin also acts on its membrane receptors (MT1/MT2) to stimulate cascading events which increase the transcriptional activity of antioxidant enzymes, suppresses the pro-oxidant enzymes, and reduce the toxic cytokines [18,19,20]. Thus, melatonin treatment protects ovaries from oxidative stress during ovulation [21] and delays the process of ovarian aging [22,23,24]. In addition, melatonin reduced oxidative DNA damage and balanced the expressions of apoptosis genes Bcl-2 and Bax [24]; therefore, it protected oocytes from oxidative stress [25,26].

Numerous studies have investigated the functions of melatonin on female reproduction [27,28,29]. For example, in out-of-season breeding of New Zealand Romney composite ewes, melatonin implantation can increase the number of lambs born [11]. The *AANAT* (aralkylamine *N*-acetyltransferase) and *ASMT* (acetylserotonin *O*-methyltransferase) were reported to be present in the rat ovary; and the kinetics of these enzymes, for example the apparent K_m_ (Michaelis constant), are similar to those found in the pineal gland of this species. The observation highly supports melatonin synthesis in ovaries. The local synthesized melatonin improves the quality of oocytes [30]. Our previous studies showed that melatonin administration significantly improved uterine receptivity and promoted embryo implantation [29].

However, there are few studies that report the effects of melatonin on early pregnancy. In the current study, the effects of melatonin on early pregnancy were explored in mice by use of different time schedules of melatonin treatment.

## 2. Results

### 2.1. Effects of Melatonin on Mice Estrus

**Figure 1:**
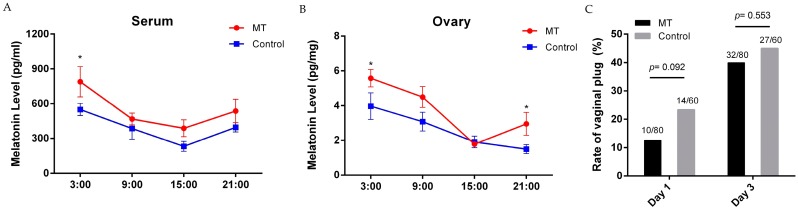
Melatonin levels and vaginal plug rate of the mice. (**A**) Serum melatonin levels at different time points (mean ± SEM, *n* = 7); (**B**) Ovarian melatonin levels at different time points (mean ± SEM, *n* = 7). Melatonin (MT): mice received 14-day melatonin treatment. Control: mice were without melatonin; (**C**) Vaginal plug rate. Day 1: the first day of the mating process. Days 1–3 represent the total accumulative vaginal plug rate from day 1 to day 3. Value *p* is based on the Pearson χ square test. “*” represents significant differences, *p* < 0.05.

The serum melatonin levels in the melatonin treatment group and in the control group at 3:00 were 790 ± 131.6 vs. 550 ± 52.79, respectively; at 9:00 they were 469 ± 51.6 vs. 386 ± 93.8, respectively; at 15:00 they were 289 ± 72.8 vs. 232 ± 42.5, respectively; at 21:00 were 538 ± 101.3 pg/mL vs. 396 ± 106.3 pg/mL, respectively. Melatonin administration elevated serum levels of mice at all-time points tested. The highest serum melatonin level occurred at 3:00 (*p* = 0.031, *n* = 7) (Figure 1A). The ovarian melatonin levels in the treatment group and in the control group at 3:00 were 5.6 ± 0.50 vs. 4.0 ± 0.77, respectively; at 9:00 they were 4.5 ± 0.60 vs. 3.1 ± 0.54, respectively; at 15:00 they were 1.9 ± 0.11 vs. 1.9 ± 0.33, respectively; at 21:00 they were 3.0 ± 0.66 pg/mg vs. 1.5 ± 0.26 pg/mg tissue, respectively. The ovarian melatonin levels in the melatonin treated group significantly increased at 3:00 (*p* = 0.31, *n* = 7) and 21:00 (Figure 1B) compared to that of the control.

Melatonin-treated mice were allowed to mate at night for three days and were evaluated for successful mating each morning. Once the females were found to have vaginal plugs, they were separated immediately. The vaginal plug rate was recorded during the period of three days. The results showed that there were no significant differences on vaginal plug rate between the control and MT-treated mice at day one or in the accumulated rate during all three days (Figure 1C).

### 2.2. Effects of Melatonin on E_2_ and P Secretion and Gene Expression in Ovaries on 0.5 Day Gestation

**Figure 2:**
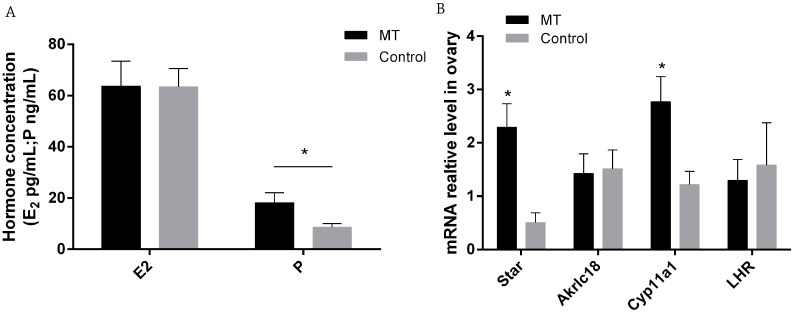
Effects of melatonin on estradiol (E_2_) and progesterone (P) levels as well as the mRNA of *StAR*, *Akrlc18*, *Cyp11a1*, and *LHR* in D0.5 gestation mice. (**A**) Serum E_2_ and P concentrations (*n* = 10); (**B**) The relative levels of mRNAs of *StAR*, *Akrlc18*, *Cyp11a1*, and *LHR* in mice ovaries (*n* = 7), detected by Q-PCR. The RT-QPCR was independently repeated three times. “*” represents a significant difference of these columns (*p* < 0.05).

P and E_2_ were measured by radioimmunoassay with 10 individuals in each group. There was no significant difference in serum E_2_ content between control and melatonin treated mice (*p* > 0.05), while P level was significantly increased in the melatonin treated group compared to the that of the control group (18.15 ± 3.96 vs. 8.60 ± 1.44 ng/mL, respectively, *p* < 0.05 Figure 2A). The results of RT-qPCR show that the expressions of two corpus luteum marker genes, *StAR* and *Cyp11a1*, were significantly higher in melatonin treated mice than those of controls (Figure 2B).

### 2.3. Effects of Melatonin on Implantation and Litter Size on 7.5 Day of Gestation Mice

**Figure 3:**
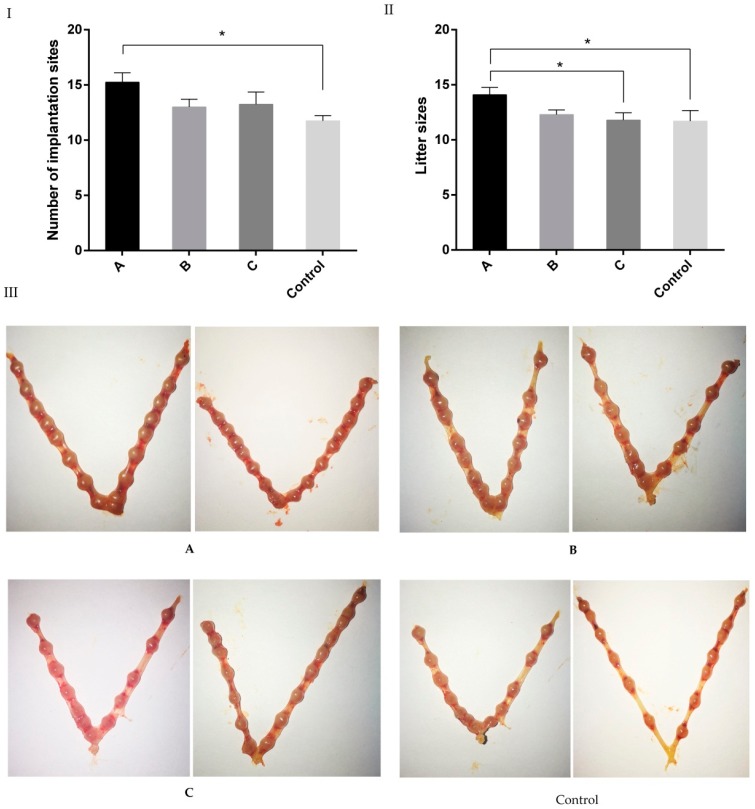
Effect of melatonin on the implantation sites and litter sizes. Mice were treated with melatonin (4.3 μg/mL) in drinking water. (**A**) Melatonin was given 14 days before mating (vaginal plugs was detected); (**B**) melatonin was given from the mating to the delivery; (**C**) melatonin was given from 14 days before mating to the delivery. (**Control**) without any treatment. *n* = 10. (**I**) Number of implantation sites; (**II**) Litter size; (**III**) Representative images of implantation sites in each group. “*” represents a significant difference between the different groups (*p* < 0.05).

The implantation sites 7.5 days after gestation were evaluated (Figure 3). The number of implantation sites in group A (15.3 ± 0.85) was significantly higher than that in the control group (11.7 ± 0.48, *p* < 0.05); the implantation sites in group B (13.0 ± 0.71) and group C (13.3 ± 1.11) were slightly higher than that of controls, however, this increase did not reach a significant difference (*p* > 0.05).

**Figure 4:**
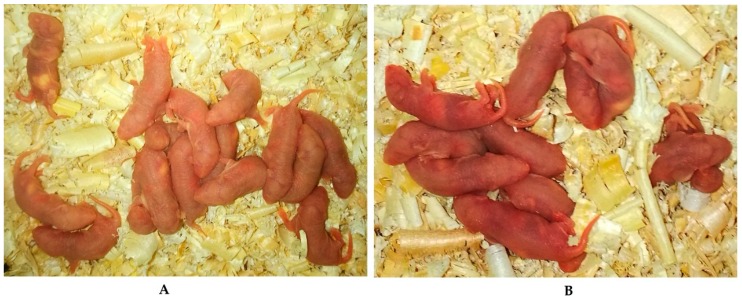

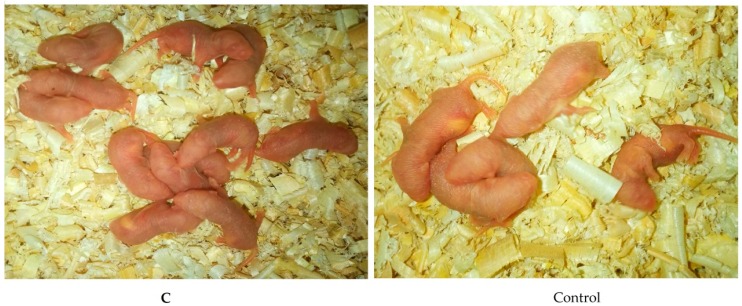
Representative images of newborn mice. (**A**) Melatonin was given 14 days before mating (vaginal plugs were detected); (**B**) Melatonin was given from the mating (vaginal plugs were detected) to the delivery; (**C**) Melatonin was given from 14 days before mating (vaginal plugs were detected) to the delivery; (**Control**) Without any treatment.

The litter size was analyzed after natural delivery. The results showed that the litter size of group A (14.1 ± 0.66) was significantly higher than that of other groups (Figure 4).

### 2.4. Effects of Melatonin on Serum E_2_ and P Levels on Day 7.5 of Early Pregnant Mice

**Figure 5:**
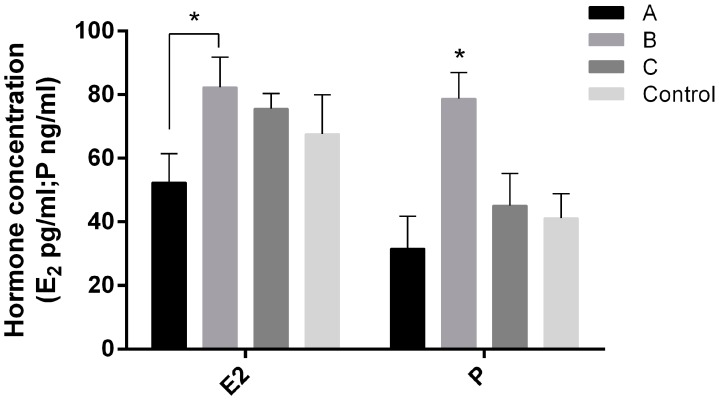
E_2_ and P concentrations in serum of Day 7.5 pregnant mice treated with or without melatonin (4.3 μg/mL) in drinking water. A, melatonin was given 14 days before mating (vaginal plugs was detected); B, melatonin was given from the mating to the delivery; C, melatonin was given from 14 days before mating to the delivery; Control: without melatonin. *n* = 10. “*” represents a significant difference of these columns (*p* < 0.05).

The results showed that E_2_ in group A (52.4 ± 9.05 pg/mL) was significantly lower than that in group B (82.4 ± 9.39 pg/mL, *p* < 0.05). P level in group B (78.82 ± 8.14 ng/mL) was significantly higher than that in other groups (Figure 5).

### 2.5. Effects of Melatonin on Gene Expressions in Mice Endometrium

**Figure 6:**
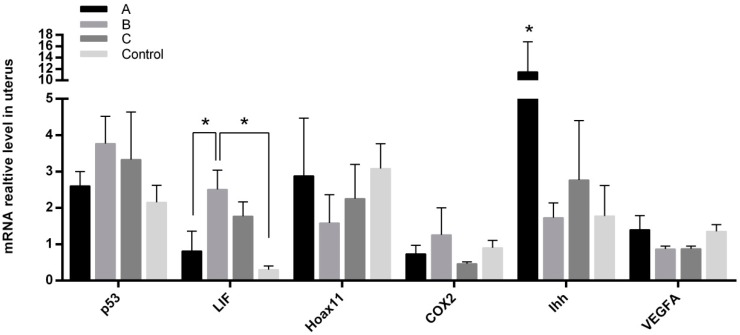
The mRNA levels of *p53*, *LIF*, *Hoxa11*, *COX2*, *Ihh*, and *VEGFA* in endometrium of 7.5 day pregnant mice. Mice were treated with melatonin (4.3 μg/mL) in drinking water. A, melatonin was given 14 days before mating (vaginal plugs were detected); B, melatonin was given from the mating to the delivery; C, melatonin was given from 14 days before mating to the delivery; Control: without melatonin. The experiment was independently repeated four times. “*” represents a significant difference of these columns (*p* < 0.05).

The gene expressions of *p53*, *Hoxa11*, *COX2*, and *VEGFA* in the endometrium of the uteri were detected among different groups. The mRNA expression levels of *p53*, *Hoxa11*, *COX2*, and *VEGFA* showed no significant differences among the groups. The expression level of *LIF* in group B was significantly higher than that in group A and in controls (*p* < 0.05); in contrast, the expression of *Ihh* in group A was significantly higher than that of other groups (Figure 6).

## 3. Discussion

Melatonin has been effectively used to combat oxidative stress, inflammation, cellular apoptosis, and to restore many tissues’ functions. The activities of melatonin are related to its pleiotropic functions. These include its direct antioxidant cascade reactions and its stimulation of antioxidant enzymes while suppressing pro-oxidant enzymes via membrane receptor (MT1/MT2) signal transduction [20]. In the current study, the effects of melatonin on early pregnant mice were investigated. It was reported that, in rats, the melatonin levels alternated periodically in the estrous cycle, the highest melatonin levels occurred during the evenings of metestrus and diestrus, while the minimum levels were detected in the evening of estrus [31]. Melatonin directly affects ovarian function to serve as a local regulator [32,33]. Many studies have tested the effects of melatonin on animal reproduction [34,35,36], and in the majority of these studies, melatonin was given either by intraperitoneal injection or implantation [37,38]. In the current study, the melatonin delivery route was through drinking water. Since the majority of water consumption by mice occurs during the night [39], the melatonin delivered though drinking water more or less resembled the physiological melatonin circadian rhythm. This aspect might render additional benefits of melatonin in reproductive physiology compared to other delivery methods. Indeed, in the study, we observed a significantly elevated melatonin level in serum as well as in the ovarian tissue in mice treated with melatonin in their drinking water compared to that of controls (Figure 1A,B). This melatonin elevation is similar to its physiological circadian rhythm. This was due to the melatonin delivered in drinking water since majority of the water was consumed by mice during the night. It was observed that only small portion of the exogenously supplied melatonin reached the ovaries. This demonstrated a high melatonin metabolism rate in animals [17]. Melatonin added to drinking water did not affect vaginal plug rate of mice, but significantly improved the implantation sites and litter size, especially in the mice given melatonin before mating. 

The results revealed that early melatonin treatment significantly upregulated the expression of the two corpus luteum marker genes, *StAR* and *Cyp11a1*, in ovaryies [40,41]. *Cyp11a1* protein localizes to the mitochondrial inner membrane and catalyzes the conversion of cholesterol to pregnenolone, the first and rate-limiting step in the synthesis of the steroid hormones. The function of *StAR* is to transport large amounts of cholesterol from the outer to inner mitochondrial membrane [42]. Both *StAR* and *Cyp11a1* were upregulated under melatonin treatment as early as on the 0.5 day of gestation. Since these genes involve pregnenolone formation, it was not a surprise that the level of P was also significantly increased with melatonin treatment in Experiment I. Ovarian P and E_2_ are essential for successful implantation in mice [1]. The P can promote endometrial luminal epithelial differentiation, stromal cell proliferation, and decidualization [2]. E_2_/ERα (estrogen receptors α) are critical for blastocyst attachment, but dispensable for subsequent decidualization [43,44]. Melatonin was reported to regulate E_2_ and P in mammals [45]. The results indicated that during early pregnancy, melatonin could improve the development of corpus luteum, promote synthesis, and release of P in ovaries which is beneficial for the early pregnancy in mice of other species.

In order to find out whether melatonin also affected the post-implantation process, melatonin was given at different gestation stages as mentioned above. The results showed that melatonin given before mating significantly increased implantation sites and litter size (Figure 3) and reduced the E_2_ level at D7.5 (Figure 5). Melatonin given after mating, for example in group B, elevated the serum P level in mice. Generally, the progesterone/estradiol (P/E_2_) ratio was reduced by melatonin treatment. The P/E_2_ ratio is crucial to implantation, and it determines pregnancy outcomes of animals [46,47]. High levels of estrogen shorten the duration window for uterine receptivity [48]. It is possible that the early melatonin treatment in pregnant mice influenced the uterine receptivity mediation.

Several genes that are crucial for implantation and endometrial development were tested in the current study. These include *p53* (a regulator of *LIF*), cyclooxygenase-2 (*COX2*), abdominal-B-like Hox genes (*Hoxa11*), and vascular endothelial growth factor (*VEGFA*). It has been reported that these genes were regulated by melatonin in some tissues and organs [49,50,51,52]. However, this was not the case in our study, and we failed to find significant differences between treated and untreated groups. This may be tissue specific, and if it is, the results indicate that these genes in the uterine might not be influenced by melatonin in the early gestation stage. The expression level of leukaemia inhibitory factor (*LIF*) in melatonin treated animals, for example in group B, was significantly higher than that of the control group. Epithelial *Ihh* is a paracrine growth factor for stromal cells and involves epithelial–mesenchymal signaling transduction [53,54]. The significantly increased expression of *Ihh* with melatonin treatment before mating suggests that the improved uterine receptivity was via the *Ihh* signaling pathway. Collectively, the results indicate that early melatonin treatment before gestation promotes the development of corpus luteum after ovulation and regulates the differentiation and secretion of E_2_ and P. The increased ratio of P/E_2_ mediates endometrial development and improves endometrial receptivity.

## 4. Materials and Methods

### 4.1. Animals

All CD-1 mice (6 weeks old) were purchased from Vital River Laboratories Co., Ltd. (Beijing, China). Mice were housed at a temperature of 22–26 °C with a 12:12 light:dark cycle (darkness from 20:00 to 08:00) and had access to food and water ad libitum. All experimental procedures were approved by the Animal Welfare Committee of China Agricultural University (Permission Number: SYXK(Beijing)2015002; The period of valid days: 22 September 2015–22 September 2020). After a week of acclimation, those mice in good condition were randomized for the following experiments.

### 4.2. Experiment Designs

Female mice (body weight 27–30 g) were treated with melatonin in drinking water (4.3 μg/mL) for 7 days. The mice had free access to water and food ad libitum. The water consumption was 5.5 to 7.0 mL/mouse/day (roughly, melatonin 0.96 μg/g body weight). Blood samples were collected from the caudal vein at 3:00, 9:00, 15:00, and 21:00.

Estrus synchronization and mating: Female and male mice were placed into the same cage at 19:00 on day 1 of the estrous cycle and separated the next morning. Then, this process was repeated for the second and the third night. Successful mating was confirmed by the presence of a vaginal plug.

Experiment I: The mice were randomly divided into two groups (80 mice/group). Mice without treatment served as the control group while the other mice were treated with melatonin in their drinking water (4.3 μg/mL); the amount was based on our previous experimental results [24]. After 14 days of melatonin treatment, spontaneous estrus female mice from both control and MT groups were carefully selected. To ensure the consistency of gestation time, female mice were mated with male mice at night. On the following morning, females were examined for vaginal plugs and sacrificed by cervical dislocation after 12 h pregnancy (D0.5). Their blood, uterine, and ovaries were collected for the subsequent assays.

Experiment II: The mice were randomly divided into four groups (80 mice/group). Mice without treatment served as the control group while the other mice were treated with melatonin (4.3 μg/mL) as follows: 14 days before breeding (vaginal plugs was detected) as group A; mice treated with melatonin from the mating to the delivery as group B; mice treated with melatonin from 14 days before mating until the delivery as group C. Ten mice from each group (vaginal plugs were detected) were sacrificed by cervical dislocation in D7.5 pregnancy. Their blood and ovaries were collected for the subsequent assays. The other pregnant mice were kept for litter size calculation after delivery.

### 4.3. The Assays of Melatonin, Estradiol-17β, and Progesterone

The serum was separated from blood samples by centrifugation at 4 °C (800× *g*) for 10 min at room temperature, then serum was collected and stored at −80 °C for melatonin radioimmunoassays (RIAs). The procedures followed were from the instructions of the Melatonin Research RIA Kit (LDN GmbH & Co., KG, Nordhorn, Germany). The ovaries were weighed and homogenized immediately in 1 mL methyl alcohol and centrifuged at 12,000 rpm for 10 min at 4 °C. The supernatant was collected and dried by Pressure Blowing Concentrator, and then the residue was reconstituted in 100 μL double distilled water for RIA assays. The recovery of melatonin from the ovaries ranged from 81.7% to 93.1%.

On days 0.5 and 7.5 of pregnancy, mice from the Experiment I and Experiment II, respectively, were anesthetized with 2% pentobarbital sodium (0.15 mL/each) intraperitoneal injectionand the blood of each mouse (melatonin-treated and control group) was collected from the caudal vein and placed at 4 °C for 30 min and then centrifuged for 10 min at 4 °C (800× *g*). The serum was collected and stored at −80 °C for future estradiol-17β (E_2_) and progesterone radioimmunoassays (RIA). The procedures followed were from the instructions of the Progesterone Direct RIA Kit (ICN Biomedicals, Irvin, CA, USA) and the Estradiol-17b (E_2_) Direct RIA Kit (ICN Biomedicals). The RIAs involved chromatographic separation of the steroids to enhance specificity. The RIAs used a tracer and a charcoal–dextran method to separate the bound and free steroid. The kits included a specific antibody that had no significant cross-reactivity with other steroids.

### 4.4. Implantation Sites Counting

On day 7.5 of pregnancy, the mice (from the Experiment II) were given 2% pentobarbital sodium (0.15 mL/each) (intraperitoneal injection) and sacrificed by cervical dislocation. The uteri were collected and cleaned, and these uteri were placed on white paper for implantation site counting and recording. 

### 4.5. Gene Expression Assay by Real-Time Q-PCR

Ovarian and uterine samples from mice of D0.5 and D7.5 of pregnancy were collected. The expression levels of cytochrome P450 family 11 subfamily A member 1 (*Cyp11a*), steroidogenic acute regulatory protein (StAR), luteinizing hormone receptor (*LHR*), and aldo-keto reductase family 1 member C18 (*Akrlc18*) genes in ovaries and the expression levels of transformation related protein 53 (*P53*), cyclooxygenase-2 (*COX2*), homeobox A11 (*Hoxa11*), leukaemia inhibitory factor (*LIF*), vascular endothelial growth factor (*VEGFA*), and indian hedgehog (*Ihh*) in uterine samples of 7.5 day pregnant mice were measured. Total RNA was extracted using the TRIzol reagent (Invitrogen Inc., Carlsbad, CA, USA) and immediately was reversely transcribed using PrimeScript™ RT reagent Kit with gDNA Eraser (TaKaRa Bio Inc., Tokyo, Japan). The expression levels of genes were detected by real-time PCR (Table 1). The reaction solution consisted of 10 μL SYBR Green, 30 mmol/L forward and reverse primers, 2 μL template, and ddH_2_O to a total volume of 20 μL. The procedure was as follows: 95 °C for 10 min; 35 cycles of 95 °C for 10 s, and 60 °C for 10 s; melting curve from 65 to 95 °C, increments of 0.5 °C every 5 s. Normalization was performed using the housekeeping gene glyceraldehyde-3-phosphate dehydrogenase (*GAPDH*). Primer sequences are listed in Table 1. Relative mRNA expression was calculated by the 2^−ΔΔ*C*t^ method.

### 4.6. Statistical Analyses

Data are expressed as mean ± SEM (standard error of mean). Statistical analyses were done using the one-way analysis of variance (ANOVA) with the aid of SPSS 19.0 statistical software followed by the Student *t*-test. *p* < 0.05 was considered significant, and *p* < 0.01 was considered highly significant.

## 5. Conclusions

In conclusion, exogenous melatonin promotes the development of corpus luteum after ovulation by up-regulating the expression of *StAR* and *Cyp11a1*. Melatonin delivered by drinking water is an effective way to enhance the physiological melatonin circadian rhythm. As a result, it increases the expression of genes involved in pregnenolone synthesis in ovary and endometrium *Ihh* expression, which mediates endometrial development and promotes embryo implantation.

## Figures and Tables

**Table 1:** Primers for RT-PCR.

Genes	Primer Sequence (5′–3′)	Tm (°C)	Accession Number
*GAPDH*	Forward: CCTGGAGAAACCTGCCAAGTAT	60	XM_017321385
	Reverse: GGAAGAGTGGGAGTTGCTGTTG		
*Akr1c18*	Forward: TTGGTCAACTTCCCATCGTC	59	NM_134066
	Reverse: GCCCTGCATCCTTACACTTC		
*P53*	Forward: TGAGGTTCGTGTTTGTGCCTGC	60	NM_001127233
	Reverse: CCATCAAGTGGTTTTTTCTTTTGC		
*StAR*	Forward: CCTTGGGCATACTCAACAACC	60	NM_011485
	Reverse: CCACATCTGGCACCATCTTACTT		
*Cyp11a1*	Forward: GGGCAGTTTGGAGTCAGTTTAC	60	NM_001346787.1
	Reverse: TTTAGGACGATTCGGTCTTTCTT		
*LHR*	Forward: CTGAGGAGATTTGGTTGCTGTA	60	NM_013582
	Reverse: ATTTGGGTGGACTTTTTTGGGG		
*MT2*	Forward: GGTGGCTCCCATGCTATCTA	59	NM_008630
	Reverse: AAACAAATTACCTGCGTTCCG		
*VEGFA*	Forward: GAGAAGACAGGGTGGTGGAAG	59	NM_001025250
	Reverse: GAAGGGAAGATGAGGAAGGGT		
*Hoxa11*	Forward: ATAGCACGGTGGGCAGGAACG Reverse: AGTCGGAGGAAGCGAGGTTTT	62	NM_010450
*Ihh*	Forward: CTACAATCCCGACATCATCTTCAA Reverse: CGGTCACCCGCAGTTTCA	62	NM_001313683
*COX2*	Forward: ACCTGGTGAACTACGACTGCTA Reverse: CCTGGTCGGTTTGATGTTACTG	59	YP_00168670
*LIF*	Forward: CTGACACCTTTCGCTTTCCTC Reverse: ACTTTCCACCTGTTTGTTCTGC	60	NM_001257135

*GAPDH*, glyceraldehyde-3-phosphate dehydrogenase; *Akr1c18*, aldo-keto reductase family 1 member C18; *p53*, transformation related protein 53; *StAR*, steroidogenic acute regulatory protein; *Cyp11a1*, cytochrome P450 family 11 subfamily A member 1; *LHR*, luteinizing hormone receptor; *MT2*, melatonin membrane receptor 2; ; *VEGFA*, vascular endothelial growth factor; *Hoxa11*, homeobox A11; *Ihh*, Indian hedgehog; *COX2*, cyclooxygenase-2; *LIF*, leukaemia inhibitory factor.

## Abbreviations

MT	Melatonin
AANAT	Aralkylamine *N*-acetyltransferase
ASMT	Acetylserotonin *O*-methyltransferase
HPG axis	Hypothalamus-pituitary-gonadal axis
K_m_	Michaelis constant
Q-PCR	Quantitative PCR
E_2_	Estradiol-17β
P	Progesterone
RIA	Radioimmunoassays
GAPDH	Glyceraldehyde-3-phosphate dehydrogenase
Akr1c18	Aldo-keto reductase family 1 member C18
p53	Transformation related protein 53
StAR	Steroidogenic acute regulatory protein
Cyp11a1	Cytochrome P450 family 11 subfamily A member 1
LHR	Luteinizing hormone receptor
VEGFA	Vascular endothelial growth factor
Hoxa11	Homeobox A11
Ihh	Indian hedgehog
COX2	CYCLOOXYGENASE-2
LIF	Leukaemia inhibitory factor
